# Tranexamic acid needs to be implemented in mass casualty incident protocols

**DOI:** 10.1007/s00068-024-02517-8

**Published:** 2024-05-27

**Authors:** Rafael Castro-Delgado, Gracia Garijo-Gonzalo, Tatiana Cuartas-Alvarez

**Affiliations:** 1 Faculty of Medicine and Health Sciences, Department of Medicine, University of Oviedo, Julián Clavería, 6, 33006 Oviedo, Spain; 2 Health Research Institute of the Principality of Asturias (Research Group On Prehospital Care and Disasters, GIAPREDE), Health Service of the Principality of Asturias (SAMU-Asturias), Oviedo, Spain; 3 RINVEMER-SEMES (Research Network On Prehospital Care-Spanish Society of Emergency Medicine), Madrid, Spain; 4 Emergencias Osakidetza. Vasc Country, Madrid, Spain

**Keywords:** Mass casualty incidents, Triage systems, Tranexamic acid, Emergency medical services

Mass casualty incidents (MCI) are a challenge for emergency medical services (EMS). There are multiple factors, both internal and external, that intervene on how they are solved. These factors cover aspects such as the use of specific protocols, coordination among different organizations, or the risks inherent to the incident itself [1]. All of this means that, although our main objective is patient care, there is little clinical research carried out in this regard for different reasons, among which we can remark on the difficulty of obtaining clinical data in a chaotic situation that, by definition, exceeds the response capacity [2]. There are few epidemiological studies that attempt to analyze the characteristics and prevalence of MCI [3, 4], and some of them for specific types [5], but there are some common elements that directly affect the clinical care of patients: a high number of patients that exceed the response capacity of the system, difficulties for clinical management in an unstable situation and long prehospital times due to access, and coordination difficulties together with lack of necessary resources[6].

From a clinical point of view, triage systems are tools that allow health professionals to establish health care based on clinical priorities, so that the most serious patients are prioritized over other milder ones[7]. Among seriously ill patients, those with uncontrolled bleeding require very specific prehospital clinical management, mainly based on early identification, hemorrhage control techniques, and rapid transport to surgical facility. Even though clinical care to these patients in MCI is usually delayed due to the chaotic situation, overwhelmed resources, and an initial lack of ambulances, it is necessary to quickly identify these patients in order to manage them as early as possible and transport them to a surgical health facility.

Some triage systems have developed the so-called “red surgical patient,” defined as a patient who requires rapid transport to the surgical facility because of uncontrolled bleeding suspicion. Early identification and rapid transport of these patients make us think that these two actions are key to reducing their mortality rate. This triage system, called META (prehospital advanced triage model)[8–10], also includes the implementation of simple techniques called “essential intervention for rapid evacuation” to be performed prior to transporting the patient, such as tourniquet or pelvic ring for bleeding control, complementing it with early hospital trauma team activation. This system was successfully used in Barcelona terrorist attacks[11], a type of event that may increase in the coming years[5].

Given that in the management of MCI, there have been few actions based on the best possible clinical evidence due to the difficulty in carrying out research studies^2^,[12], we must integrate the scientific knowledge that arises in other fields to improve prehospital response in these cases[13]. We must take into account that our main objective with all the actions we carry out is to reduce the critical mortality rate.

It is very well known that bleeding patients benefit from shorter prehospital times[14, 15], and that prehospital times in MCI affect triage decisions[16]. That said, the use of tranexamic acid has been shown to reduce the mortality of trauma patients with hemorrhage[17], both cerebral[18] and other types. Its intramuscular application has good absorption[19], and has been recommended in certain situations[20] based on previous experiences related to terrorist attacks, like the one in Manchester in 2017[21]. Even in hemorrhagic shock in swine models, intramuscular administration provides serum concentrations associated with the inhibition of fibrinolysis[22], and in bleeding trauma patients, it is well tolerated and rapidly absorbed[23]. This is a very important aspect to be considered in MCI, as it would make its administration much easier. For this reason, and given that most EMS have internal response protocols for MCI, many of them based on similar concepts, we consider that tranexamic acid should be included within the essential rapid actions at the prehospital stage to be performed on severe patients with suspected uncontrolled hemorrhage, who should be identified early and quickly transferred to a surgical center (Fig. 1). The decision to include tranexamic acid in MCI protocols would not imply assuming great clinical risks for the patient due to the low rate of complications[24], even lower because the patient would have a prior clinical evaluation, and the economic impact would be irrelevant. It may be recommended to adapt its commercial presentation to prefilled syringes (5 ml/1 g) to facilitate intramuscular administration in MCI and its implementation in the clinical and logistic material. This logistical need linked to the MCI protocols would mean that TXA ambulance stocks should be updated with enough doses for a proper MCI response, or even that it should be implemented in other non-clinical first responders that may be authorized to administer it.

**Fig. 1:**
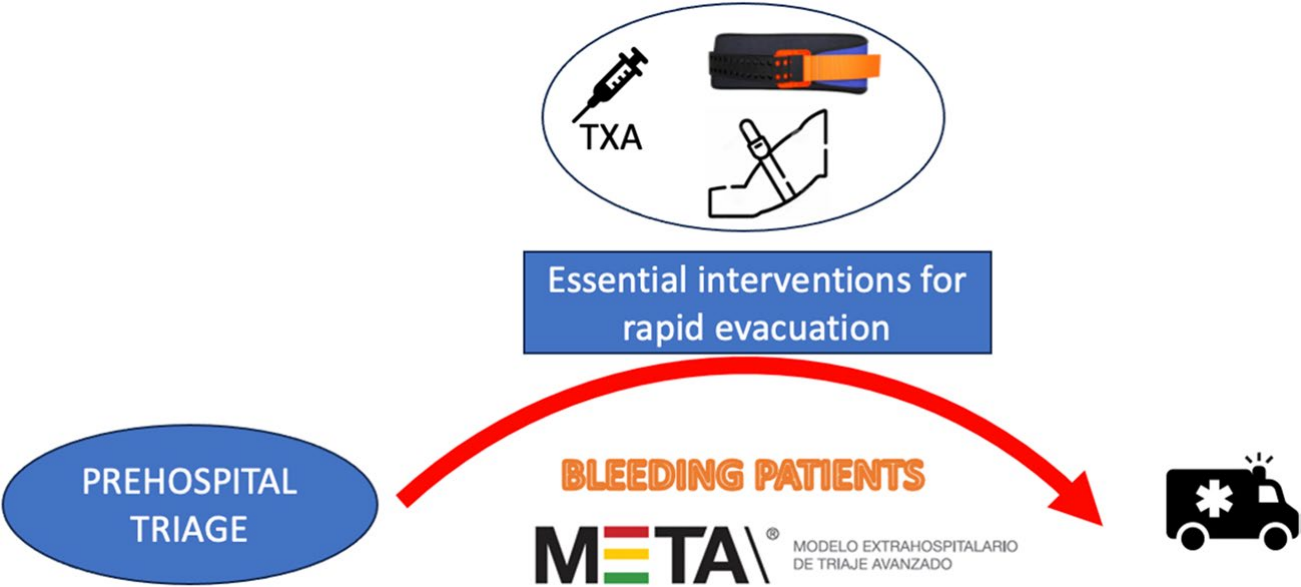
Essential prehospital interventions for bleeding patients in mass casualty incidents using the META triage system

Early use of intramuscular tranexamic acid in MCI in selected patients, those with high suspicion of uncontrolled hemorrhage, could reduce the mortality in this group of patients, who are our main target when organizing prehospital care in MCI and for whom little can be done in the prehospital setting apart from rapid identification, bleeding control actions, and quick transport to a proper surgical facility. For this reason, we believe that it should be integrated worldwide into the EMS protocols for MCI, probably in different ways (when, who, how) depending on the protocol, and administered as soon as possible by an authorized health care provider in selected patients, those with clinical suspicion of uncontrolled hemorrhage. Once this is done, more studies should be carried out to analyze the impact of this decision, being aware of the difficulty of carrying them out at a prehospital level during an MCI. In this sense, trauma registries including prehospital data and MCI identification may help to achieve this research goal.

## Data Availability

No datasets were generated or analyzed during the current study.
